# Understanding Resistance Mechanisms and Expanding the Therapeutic Utility of PARP Inhibitors

**DOI:** 10.3390/cancers9080109

**Published:** 2017-08-22

**Authors:** Joline S. J. Lim, David S. P. Tan

**Affiliations:** 1 Department of Hematology-Oncology, National University Cancer Institute of Singapore, National University Hospital, Singapore 119228, Singapore; joline_sj_lim@nuhs.edu.sg; 2 Cancer Science Institute Singapore, Women’s Cancer Research Group, Singapore 117599, Singapore

**Keywords:** PARP inhibitors, targeted therapy

## Abstract

Poly-(ADP-ribose) polymerase (PARP) inhibitors act through synthetic lethality in cells with defects in homologous recombination (HR) DNA repair caused by molecular aberrations such as BRCA mutations, and is approved for treatment in ovarian cancer, with promising clinical activity against other HR defective tumors including breast and prostate cancers. Three PARP inhibitors have been FDA approved, while another two have shown promising activity and are in late stage development. Nonetheless, both primary and secondary resistance to PARP inhibition have led to treatment failure, and the development of predictive biomarkers and the ability to identify and overcome mechanisms of resistance is vital for optimization of its clinical utility. Additionally, there has been evidence that PARP inhibition may have a therapeutic role beyond HR deficient tumors which warrants further investigation, both as single agent and in combination with other therapeutic modalities like cytotoxic chemotherapy, radiation, targeted therapy and immunotherapy. With new strategies to overcome resistance and expand its therapeutic utility, PARP inhibitors are likely to become a staple in our armamentarium of drugs in cancer therapeutics.

## 1. Introduction

The emergence of targeted therapy at the turn of the century has led to a paradigm shift in cancer therapeutics from a “one size fits all” strategy to one with an emphasis on precision medicine. Drugs are now being designed specifically to exploit molecular aberrations found in tumors, thus maximizing therapeutic efficacy while minimizing systemic toxicity.

It has been more than 35 years since the role of adenosine-diphosphate(ADP)-ribose and its effects was first described by Durkacz and colleagues [1], and our understanding of poly-(ADP-ribose) polymerase (PARP) inhibition has greatly expanded since. PARP inhibitors profoundly sensitize cancer cells to DNA damaging agents and act through synthetic lethality in cells with defects in homologous recombination (HR) DNA repair caused by molecular aberrations such as *BRCA1/2* mutations [2]. Olaparib was the first PARP inhibitor to be approved by the US Food and Drug Administration (FDA) as monotherapy in a third line and beyond setting for patients with deleterious or suspected germline *BRCA1/2*-mutant advanced ovarian cancer, and by the European Medical Agency (EMA) as monotherapy maintenance therapy for patients with platinum-sensitive, relapsed germline or somatic *BRCA1/2*-mutant epithelial ovarian, fallopian tube or primary peritoneal cancers who have responded to platinum-based chemotherapy. Subsequently, rucaparin and niraparib have also been FDA approved for similar indications, and PARP inhibition has shown clinical activity against other HR defective tumors including breast and prostate cancers [3,4].

Nonetheless, in the majority of patients, resistance to PARP inhibition inevitably develops, leading to treatment failure. Additionally, a proportion of patients exhibit primary resistance to these drugs despite harbouring genomic features of DNA repair deficiency. Contrarily, there are tumors without known mutations in DNA damage repair genes that are also sensitive to and may benefit from PARP inhibitors [5]. In this respect, the development of predictive biomarkers and the ability to identify and overcome mechanisms of resistance will be crucial to enable further optimization of tis clinical utility. Furthermore, recent preclinical data and early clinical studies suggest that there is scope to enhance the efficacy of PARP inhibitors and extend the clinical utility of this class of compounds beyond DNA repair deficient tumors. 

In this review, we will discuss the mechanisms of action of PARP inhibitors, detail the current status of PARP inhibitors that are currently FDA approved or have shown promising activity in clinical studies, and explore the mechanisms of resistance to PARP inhibition and potential approaches to overcome them, including combination strategies for treatment.

## 2. DNA Damage Repair (DDR) and Mechanisms of Action of PARP Inhibition

**Figure 1:**
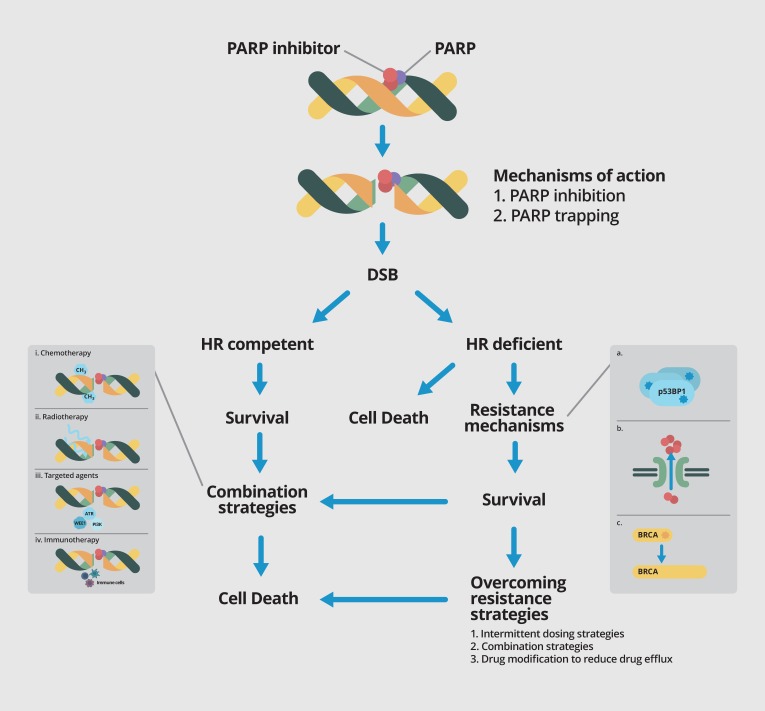
PARP inhibitors cause DNA DSB (double strand break) by via inhibition of PARP enzyme activity and PARP trapping. In HR (homologous recombination) competent tumors, tumor cells with intact homologous recombination repair will be able to survive. However, in *BRCA1/2* mutant and other HR deficient cancers that are reliant on base-excision repair based on the PARP pathway, blockade of this pathway by PARP inhibition leads to synthetic lethality and cell death. Multiple resistance mechanisms against PARP inhibitors have been elucidated, including somatic mutations in p53BP1 (**a**); upregulation of drug efflux transporters such as PgP (**b**); and somatic mutations in BRCA gene leading to restoration of the open reading frame and thus BRCA function (**c**). Strategies to overcome resistance include intermittent dosing, combination strategies and drug modification to reduce drug efflux. Various combination strategies are currently underway to further exploit the role of PARP inhibitors, including combination with chemotherapy (**i**); radiation therapy (**ii**); targeted agents (**iii**) and immunotherapy (**iv**).

DNA damage involving single and double strand breaks, occur as part of routine cellular response to environmental and metabolic impact on cellular tissue. Single-strand breaks (SSBs) on DNA activates PARP, which then binds onto DNA and activates the C-terminal domain, initiating a series of PARylation events that leads to DNA damage repair (DDR). Once DDR is completed, PARP autoPARylates and is released from the repaired DNA strand [6]. Inhibition of PARP leads to stalling of the DNA replication fork, converting SSBs to double-strand DNA breaks (DSBs). DSBs are repaired through 2 major pathways, the high fidelity HR pathway, and the more error-prone non-homologous end-joining (NHEJ) pathway [7]. Restoration of the DNA strand through the HR pathway is mediated by proteins including BRCA 1/2, 53BP1, RAD51 among others, and BRCA 1/2 protein is a major player in HR repair of DNA. The interplay between the roles of PARP and HR leads to the concept of synthetic lethality. In patients whose tumor exhibit HR deficiency, e.g., through *BRCA1/2* mutations, double-stranded DNA (dsDNA) repair is impaired, leading to cells becoming increasingly reliant on PARP as a primary mechanism of DDR and in turn, making them exquisitely sensitive to PARP inhibition [8] (Figure 1).

Emerging evidence also suggests that in addition to the catalytic action of PARP inhibitors leading to synthetic lethality in HR deficient tumors, PARP inhibitors also causes trapping of PARP1 and PARP2, forming PARP-DNA complexes with increased cytotoxicity leading to increased cell killing [9]. The potency of PARP trapping differs amongst different PARP inhibitors and does not seem to correlate with its catalytic effect, potentially accounting for differential potency in some PARP inhibitors such as talazoparib [10] (Table 1).

## 3. Currently Available PARP Inhibitors

There are currently three PARP inhibitors that have been FDA-approved for use—olaparib (Astra Zeneca, London, UK), rucaparib (Clovis Oncology, Boulder, CO, USA), and niraparib (Tesaro Inc., Waltham, MA, USA). Other PARP inhibitors in active development include veliparib (AbbVie pharmaceuticals, North Chicago, IL, USA) which obtained FDA orphan drug designation in 2016, and talazoparib (Pfizer Pharmaceuticals, New York City, NY, USA) which has shown promising phase I data (Table 1).

Olaparib was the first PARP inhibitor to be approved by the EMA and FDA authorities for treatment of patients with *BRCA1/2* mutant ovarian cancers. Olaparib was first shown in simultaneous publications from two independent groups to successfully induce cell killing effects in BRCA-deficient cancer cells through inhibition of DDR [12,13]. Phase I studies subsequently demonstrated good safety and tolerability up to a dose of 400 mg twice daily, with dose limiting toxicities of grade 4 thrombocytopenia and grade 3 somnolence, and also showed early signals of antitumor activity with a response rate of up to 46% in heavily pre-treated *BRCA1/2*-mutant cancers [14,15]. Following this, paired phase II studies were then carried out which confirmed cell killing effect and demonstrated dose response in *BRCA1/2* mutant breast and ovarian cancers, with better patient outcomes observed for patients with platinum sensitive compared to platinum resistant disease [16,17]. While the phase III trial of olaparib compared to Caelyx^®^ (liposomal doxorubicin, Janssen Pharmaceuticals, Beerse, Belgium) in ovarian cancer recurring within 12 months of platinum-based chemotherapy failed to show an improvement in progression-free survival (PFS), possibly due to greater than expected efficacy in the control arm [18], further studies of the role of olaparib as maintenance treatment in platinum-sensitive ovarian cancer following platinum-based chemotherapy demonstrated improved progression and overall survival [19,20,21], leading to its approval by the European Medicines Agency, and accelerated FDA approval for advanced *BRCA1/2*-mutant ovarian cancer. Besides ovarian cancer, olaparib has also shown potential efficacy in other cancers involving both germline and somatic *BRCA1/2* mutations, including that of prostate, breast, gastric and pancreatic cancer [3,22,23]. Most recently, the phase III OLYMPIAD study randomizing metastatic germline BRCA-mutant breast cancer patients who have progressed through two or more lines of chemotherapy to olaparib or treatment of physician’s choice has shown superior PFS with olaparib [4].

Besides olaparib, rucaparib successfully obtained FDA approval in December 2016 for treatment of patients with germline or somatic *BRCA1/2*-mutant ovarian cancers that have progressed on two or more lines of chemotherapy. A phase I study of rucaparib investigated different dosing schedules, and showed that continuous drug dosing was more effective than intermittent dosing through quantification of loss of PAR chains through peripheral blood lymphocytes [24]. The phase II ARIEL2 study confirmed that rucaparib prolonged PFS in patients with platinum-sensitive recurrent ovarian cancers, and led to FDA approval of rucarapib [25]. Interestingly, the ARIEL2 study included patients with germline *BRCA1/2*-wild type platinum sensitive recurrent ovarian cancers, but utilized a next generation sequencing loss of heterozygosity (LOH) assay that as a biomarker for HR deficiency in *BRCA1/2*-wild type platinum sensitive ovarian cancers. The hypothesis was that the extent of genome wide loss of heterogeneity would be able to predict for response to PARP inhibition. As predicted, *BRCA1/2*-mutant cancers had improved response (80% vs. 10%) and PFS compared to LOH low subgroup (hazard ratio (HR) 0.27, *p* < 0.0001). The LOH high subgroup also had improved RECIST response (29% vs. 10%) and longer duration of response (10.8 months vs. 5.6 months, *p* = 0.022) compared to LOH low subgroup, although median PFS between the two subgroups were similar (5.7 months vs. 5.2 months). A further planned post-hoc analysis subsequently showed that a cut off of 16% compared to 14% for the LOH assay may be a better predictor of PFS [26], and this is currently being validated in the ARIEL3 study (NCT01968213) which investigates the use of rucaparib as maintenance therapy in platinum sensitive ovarian cancer. 

The latest PARP inhibitor to be approved by the FDA is niraparib, based on the phase III NOVA study that investigated the role of this PARP 1/2 inhibitor as maintenance therapy for patients with platinum-sensitive, recurrent ovarian cancer. In this study, patients with platinum-sensitive disease were included regardless of germline *BRCA1/2* mutation and HR deficiency status, while results were stratified to investigate role of HR deficiency biomarkers for response [27]. Definition of HR deficiency was determined by the myChoice HRD test, a scoring system incorporating loss of heterozygosity (LOH), telomeric allelic imbalance (TAI) and large-scale state transitions (LST) developed by Myriad Genetics (Salt Lake City, UT, USA), and validated in patients undergoing neoadjuvant treatment for triple negative breast cancer with platinum, gemcitabine and iniparib. Interestingly, on top of PFS, this score was also found to predict for pathologic complete response rates [28]. Improvement in PFS was observed regardless of germline *BRCA1/2* mutation or HR deficiency status, although improvement in PFS was most marked in the germline *BRCA1/2* mutant group (HR 0.27, confidence interval (CI) 0.17–0.41). This was followed by the HR deficient group (HR 0.38, CI 0.24–0.59) and the non-germline *BRCA1/2* mutant cohort (HR 0.45, CI 0.34–0.61) [27]. 

Veliparib has been granted FDA orphan drug designation in 2016 based on a phase II BROCADE study in breast cancer that showed improved response rate (77.8% vs. 61.3%) when veliparib was combined with carboplatin and paclitaxel. although there was no difference in PFS. An ongoing phase III BROCADE 3 study will further verify the phase II findings. Besides *BRCA1/2* mutant cancers, veliparib has also been tested in other cancers such as non-small cell lung cancer (NSCLC) and melanoma, although results have been disappointing [29,30].

Most recently, promising results have been published for talazoparib, an oral PARP inhibitor with equivalent catalytic activity compared to olaparob and rucaparib, but superior PARP-trapping capabilities that may account for its increased potency. In a phase I, two-part study of talazoparib in treatment refractory tumors, including germline *BRCA1/2* mutant and other selected sporadic cancers, single agent antitumor activity was observed in *BRCA1/2* mutation-associated breast and ovarian cancers, and also patients with pancreatic and small cell lung cancers whose tumors harbor genomic aberrations involving DNA repair mechanisms such as PALB2 mutations [31]. 

## 4. Understanding the Mechanisms of Resistance to PARP Inhibitors

Since the emergence of targeted therapy as a modality of treatment in cancer, the understanding of resistance mechanisms has become vital in development of new strategies to overcome resistance and resensitise tumor cells to therapy (Figure 1). 

One of the first mechanisms of resistance to PARP inhibition that was discovered was that of restoration of *BRCA1/2* function through mutations that lead to restoration of open reading frames (ORFs) of the gene. Studies from independent groups have shown that restoration of *BRCA1/2* function occurred in cell lines treated with PARP inhibitors through secondary mutations that restored the ORF through formation of new isoforms encoding the RAD51 binding domain at the C-terminus [32]. This was subsequently verified in tumor samples from patients with *BRCA1/2*-mutant ovarian cancers, and also *BRCA1/2*-mutant breast cancers. Interestingly, such mutations were observed not only in patients with *BRCA1/2*-mutant breast cancers that were resistant to PARP inhibitors, but also patients who had platinum resistant disease [33]. A study involving ovarian cancer patients treated with carboplatin showed that one out of 60 patients (1.7%) harbored a *BRCA1/2* mutation that restored ORF in one cohort of patients, while in another cohort, the incidence of such mutations was 46.2% in patients who had platinum-resistant disease, significantly higher when compared to 5.3% in patients with platinum-sensitive disease (*p* = 0.003) [34]. 

Besides mutations that restore the ORF of the RAD51 binding domain, development of resistance could also occur due to partial restoration of HR through somatic loss of *53BP1*, a mechanism that is unique to *BRCA1* mutations and not *BRCA2* deletions [35]. The 53BP1 protein is a NHEJ factor that when deleted, promotes damaged DNA ends to produce recombinogenic ssDNA competent for HR [36,37]. Other additional factors that are implicated with 53BP1-mediated PARP resistance include that of the RAP1-interacting factor 1 (RIF1) and RNF8 ubiquitin ligase, which together with 53BP1, regulate HR in *BRCA1/2* mutant cells. When 53BP1 function is lost, suppression of 53BP1 led to decreased NHEJ and compensatory increased HR mediated DNA repair [38,39,40].

A third mechanism of resistance involves that of pharmacological resistance through multidrug efflux transporters such as P-glycoprotein (Pgp). Pgp is encoded by the MDR1 gene, and upregulation of Pgp expression has been known to be a mechanism of resistance to chemotherapy [41]. In *BRCA1/2* mutant cancers that have been treated with PARP inhibitors, increased expression of *MDR1* genes have been observed, leading to increased expression of Pgp and a resultant higher rate of drug efflux, diminishing the therapeutic intracellular effect of PARP inhibitors [42]. 

## 5. Overcoming PARP Resistance 

Knowledge regarding the aforementioned resistance mechanisms to PARP inhibitors will facilitate the development of strategies to overcome them. Tumor cells undergo complex evolutionary mechanisms not unlike the Darwinian evolution of species, and exploitation of such evolutionary models may allow for more efficacious cell killing effect [43,44]. This could potentially be achieved through varying the dosing schedules of PARP inhibitors by intermittent or metronomic approaches. While a phase II study exploring intermittent compared to continuous dosing of rucaparib concluded that a continuous dosing of rucaparib is required for optimal response [24], intermittent dosing of PARP inhibitors in combination strategies with chemotherapy like cisplatin or caelyx was found to be more tolerable with promising antitumor activity, and would benefit from further studies to verify preliminary findings [45,46]. Besides improving tolerability, intermittent dosing of PARP inhibitors may also prevent accelerated emergence of resistant clones, allowing for a patient to benefit from a longer period of exposure to such drugs. In the context of reducing drug efflux, AZD2461, a PARP inibitor that is a poor P-glycoprotein substrate, has been developed and has shown increased response compared to olaparib in preclinical breast cancer models [47].

## 6. Expanding the Therapeutic Utility of PARP Inhibitors beyond BRCA Mutant Cancers

The presence of germline or somatic *BRCA1/2* mutations currently remain the strongest predictive biomarkers for response to PARP inhibitors, but studies have also shown that other mutations may render sensitivity to single agent PARP activity. A systemic screen of cancer cell lines with a large panel of drugs revelaed that Ewing’s sarcoma cells harboring the EWSR10FLI1 gene translocation was exquisitely sensitive to PARP inhibitors [48]. A non-randomised phase II trial that was subsequently conducted showed that treatment of metastatic Ewing’s sarcoma with olaparib was safe and well tolerated, although single agent activity appeared to be minimal [49]. Combination strategies with other drugs such as trabectadin used in treatment of Ewing’s sarcoma have showed promising results in a preclinical setting, and clinical trials are currently underway to validate these findings in patients [50]. Unsurprisingly, there is a growing interest to explore strategies that exploit potential synergisms with other therapeutic options that cause DNA damage repair in an effort to expand the role of PARP inhibitors beyond that in *BRCA1/2* mutant or HR deficient cancers. These include combinations with other cytotoxic chemotherapy, radiation, targeted agents and immunotherapeutic agents (Figure 1, Table 2).

The combination of PARP inhibitors with cytotoxic chemotherapy has focused largely on drugs that cause DNA damage repair, in the hope that this will lead to synergistic effects from the inhibition of HR by PARP inhibitors. The combination of PARP inhibitors with platinum agents has been tested in recurrent platinum sensitive ovarian cancers with the combination of olaparib plus paclitaxel and carboplatin followed by maintenance monotherapy demonstrating significantly improved progression-free survival versus paclitaxel plus carboplatin alone [51]. However, the dose of olaparib (200 mg twice a day) and carboplatin (area under the curve (AUC) 4 mg/mL per minute) had to be reduced in the combination arm due to the risk of myelotoxicity and it remains unclear if this combination strategy confers any significant benefit over standard dose platinum-based chemotherapy followed by PARP inhibitor maintenance therapy. Besides platinum salts, other DNA alkylating agents that have been tested extensively include temozolamide, which has been combined with veliparib and tested in patients with melanoma and glioblastomas (GBM), albeit with limited success [29,52]. However, the results of the combination of temozolamide and olaparib in patients with recurrent small cell lung cancer (SCLC) was recently presented, and showed promising results, with good tolerance and response rate of 48% with a median PFS of 5.6 months [53]. Nonetheless, combination strategies with chemotherapy have been limited by toxicities, predominantly that of myelosuppression, and strategies to overcome these toxicities have included the use of intermittent dosing schedules of PARP inhibitors [24].

As with DNA damaging agents, ionizing radiation has direct effects of dsDNA, causing strand breaks and replicative stress, making it an attractive combination compared to chemotherapy combinations that have been limited by toxicities requiring multiple dose reductions. Preclinical studies have demonstrated an improvement in tumor response to radiation when PARP inhibitors were introduced, possibly through induction of S phase arrest through DDR and delay in dsDNA processing by PARP inhibition, leading to further sensitisation of cells to radiation [54]. Studies in several tumor types including head and neck tumors (HNSCC), GBM and pancreatic cancers have shown that PARP inhibition is a potent radiosensitiser, enhancing the therapeutic ratio of radiation by disabling DNA replication in HR-deficient tumor cells [55,56,57]. In GBM, triplet combination of PARP inhibition, radiotherapy and temozolamide has shown further synergistic effects compared to doublet therapy of PARP inhibition and radiotherapy [58].

The combination of PARP inhibition with targeted therapies, especially other drugs involved in the DDR pathway, has generated much interest, in the hope of further exploiting the concept of synthetic lethality. Following DNA damage, cellular pathways are initiated that trigger cell cycle delay by activation of cell cycle checkpoint proteins. This cell cycle arrest represents a survival mechanism that enables tumor cells to repair their own damaged DNA, and abrogation of cell cycle checkpoints, before DNA repair is completed, can induce apoptosis and lead to cell death. Thus, inhibitors of cell cycle checkpoint proteins in cancer cells may lead to circumvention of cell cycle delay resulting in increased sensitivity to DNA-damage induced apoptosis [59]. Synergistic cytotoxicity has been described when drugs that inhibit cell cycle regulators are combined with PARP inhibition, and multiple early phase studies have been initiated where drugs inhibiting cell cycle regulators like Wee1, ATR and CHK are being combined with PARP inhibitors [60]. The combination of Wee1 and PARP inhibitors to radiosensitize pancreatic cancer cells has been tested in xenograft models, and shown to produce significant radiosensitisation with a 13-day delay in tumor volume doubling and complete eradication of 20% of tumors compared to radiation alone [61]. Similar studies looking at combination of Wee1 and PARP inhibitors in acute myeloid leukemic cells have also shown synergistic inhibition of cell growth, leading to enhanced DNA damage and induction of apoptosis [62]. Besides Wee1, targeting the ATR/CHK1 axis has also shown synergistic results in preclinical models. A study in *BRCA2* mutant ovarian cancer models showed that combination of olaparib with AZD6738 (ATR inhibitor) or MK8776 (CHK1 inhibitor) induced greater tumor regression compared to single agent therapy [63]. More recently, a preclinical study investigating the role of CHK1 inhibitor showed that the combination of LY2606368 and PARP inhibition caused increased DNA damage and cell death, likely due to impaired G2/M checkpoint inhibition [64]. Similar results have also been shown in SCLC, providing preclinical proof-of-concept supporting initiation of clinical studies for combination treatment in patients with platinum sensitive or resistant relapsed SCLC [65].

Inhibition of the PI3K/AKT/mTOR pathways in combination with PARP inhibitors have also showed synergistic activity in *BRCA1/2*-mutant breast cancers, with pharmacoydynamic studies showing corresponding downstream effects [66]. The rationale for combination could be attributed to observation of increased levels of H2AX, suggesting an accumulation of dsDNA breaks requiring PARP activity for DNA repair, possibly accounting for the exquisite sensitivity of tumor cells to doublet therapy of PARP and PI3K inhibition [67]. Besides drugs targeting the DDR pathway, studies have also suggested potential synergistic effects between PARP inhbitors and the PI3/ATK/mTOR pathways. In a preclinical study of prostate cancer, PARP inhibition apperars to trigger a p53-dependent cellular senescence in PTEN-deficient prostate cancer cell lines, and combination of PARP and PI3K inhibitors had synergistic inhibition of growth in both in vitro and in vivo models [68]. Similar studies in BRCA-wild type, PI3K-mutant triple negative breast cancer cell lines showed that combination therapy of PARP and PI3K inhibition with carboplatin blocked tumor growth in mouse xenograft models, with decrease in tumor cell proliferation and tumor induced angiogenesis [69]. Besides PI3K inhibitors, combination of PARP inhibitor and everolimus, an mTOR inhibitor, has shown similar efficacy in *BRCA1/2*-proficient triple negative breast cancers [70]. The combination is currently under active investigation in early phase studies (NCT02338622).

Combinations of antiangiogenic agents like cediranib and bevacizumab with PARP inhibitors have also been tested on the basis of preclinical studies suggesting that hypoxemic states can suppress HR due to downregulation of HR repair proteins, thus inducing “BRCAness” and sensitizing cells to PARP inhibition [71]. In ovarian cancer, a phase II study explored the combination of cediranib with olaparib in patients with recurrent platinum-sensitive ovarian cancers, and found promising data of improved PFS compared to single agent olaparib (HR 0.42, *p* = 0.0005), and this is currently being further explored in a phase III study [72]. Using a similar approach in multiple myeloma, bortezomib has been shown to induce “BRCAness” through depletion of nuclear ubiquitin and abrogation of H2AX polyubiquitylation, leading to sensitization of cells to treatment with veliparib [73]. 

There has been much interest in the combination of PARP inhibitors and immunotherapy, based on preclinical data that support the association of *BRCA1/2* mutational status with neoantigen load, tumor infiltrating lymphocytes and the expression of PD/PDL1 or CTLA4, thus forming the rationale for combination therapy. There have been data indicating that *BRCA1/2* deficient cancers express higher levels of neoantigens and are therefore likely to be more immunogenic, and preclinical studies showed that a combination of PARP inhibition with a CTLA-4 antibody showed synergistic activity in *BRCA1/2* mutant ovarian cancer [74]. In vitro and in vivo models of breast cancer have also shown that PARP inhibition inactivates of GSK3β, which in turn upregulate PD-L1 expression, providing evidence to support combination of PARP inhibitors and immune checkpoint blockade for treatment of breast cancer [75].

## 7. Conclusions

Over the past decade, convincing evidence has emerged regarding the role of PARP inhibitors in *BRCA1/2* mutant cancers including ovarian, breast and prostate cancers [3,4,16,19]. There is no doubt that PARP inhibitors have carved out a niche in the treatment algorithm of ovarian cancers and their role is being actively investigated in multiple other tumor types. There are continually emerging signals of efficacy and clinical utility in varying cancer types, and further understanding of the mechanism(s) of action and resistance has led to exploration of novel therapeutic combinations. Nonetheless, several outstanding issues still remain to be answered, that may eventually help to better define the patient populations that will benefit from treatment with PARP inhibitors.

Firstly, there is an urgent need for a consistent predictive biomarker to aid patient selection with tumors that exhibit a “BRCA syndrome”-like phenotype. Besides germline *BRCA1/2*-mutant cancers, there has been increasing evidence that PARP inhibitors may have a role in somatic *BRCA1/2*-mutant cancers, or cancers associated with HR deficiency [3,25], and more recently, possibly even tumors deficient in chromatin regulation like cancers with *ARID1A* mutations [76]. While we have had hits, there were also misses like the use of ATM loss as a predictive biomarker for response in gastric cancer [22], and the availability of a predictive biomarker equivalent to germline/somatic *BRCA1/2* mutations that can more consistently or even better predict for tumor responses across a variety of tumor types remains elusive. Secondly, while multiple combination strategies are currently being explored, the most effective means of combination in terms of sequencing of drugs and the optimal timing to introduce PARP inhibitors in a patient’s long journey of cancer treatment is still a controversial subject. More importantly, there is a need to embrace the use of novel adaptive trial design to allow for validation of mechanistic hypotheses, and to allow for cohort expansion in tumor types that show early signs of response [77]. This will allow for investigators to build upon promising combinations in a swifter fashion without administrative delays of starting up multiple trials, and ultimately improve cost-effectiveness while giving patients earlier access to drug combinations that will potentially improve their outcomes. Thirdly, a better understanding of the underlying mechanisms involved in PARP inhibitor resistance will also facilitate the development of novel therapeutic strategies to address this issue. Ultimately, by bridging these gaps in our knowledge, we envisage that the utility of PARP inhibitors will continue to expand as a therapeutic staple in our armamentarium of drugs against cancer. 

## Figures and Tables

**Table 1:** Current PARP inhibitors approved or in late stage development.

Drug	Company	IC_50_/nM	Relative PARP Trapping Potency [9,11]	Predominant Toxicities
Olaparib	Astra Zeneca	6	1	GI toxicities, fatigue, anemia
Rucaparib	Clovis	21	1	GI toxicities, fatigue, anemia, liver dysfunction
Niraparib	Tesaro	60	~2	Myelosuppression, GI toxicities, fatigue
Veliparib	AbbVie	30	<0.2	Fatigue, alopecia, GI toxicities, myelosuppression
Talazoparib	Pfizer	4	~100	GI toxicities, fatigue, lymphopenia

**Table 2:** Selected ongoing trials of combination strategies with PARP inhibition.

Drug	PARP Inhibitor	Phase	Tumor Type	NCT
*Cytotoxic*				
*Platinums*				
Carboplatin	Olaparib	I	Solid tumors	02418624
Carboplatin + paclitaxel	Talazoparib	I	Solid tumors	02317874
Carboplatin + paclitaxel	Veliparib	III	Breast	02163694
Carboplatin + etoposide	Veliparib	II	SCLC	02289690
Carboplatin + gemcitabine	Veliparib	II	Germ cell	02860819
Carboplatin + paclitaxel + avastin	Olaparib	III	Ovarian	02477644
Cisplatin	Veliparib	II	Breast	02595905
Cisplatin + gemcitabine	Talazoparib	I	Solid tumors	02537561
*Temozolomide-based*				
Irinotecan +/− temozolomide	Talazoparib	I	Paediatric tumors	02392793
Temozolomide or irinotecan	Niraparib	I	Ewing’s sarcoma	02044120
Temozolomide + capecitabine	Veliparib	I	PNET	02831179
5FU-based				
FOLFOX	Veliparib	I/II	Pancreas	0149865
FOLFIRI	Veliparib	II	Pancreas	02890355
*Others*				
Liposomal irinotecan	Veliparib	I	Solid tumors	02631733
Decitabine	Talazoparib	I	AML	02878785
*Radiation*				
RT	Olaparib	I	HNSCC	02229656
RT	Olaparib	I	Breast	02227082
RT	Olaparib	I	Esophagus	01460888
RT	Olaparib	I	Sarcoma	02787642
RT +/− cisplatin	Olaparib	I	NSCLC	01562210
RT + carboplatin + paclitaxel	Veliparib	I/II	NSCLC	01386385
Rd223	Niraparib	I	Prostate	03076203
*Targeted therapy*				
*Cell cycle check point inhibitors*				
AZD1775 (Wee1)	Olaparib	I	Solid tumors	02511795
Prexasertib (CHK1)	Olaparib	I	Solid tumors	03057145
VX-970 (ATR) + cisplatin	Veliparib	I	Solid tumors	02723864
Dinaciclib (CDK)	Veliparib	I	Solid tumors	01434316
*Anti-angiogenics*				
Cediranib (VEGF)	Olaparib	II	Ovarian; GBM; solid tumors;	02345265; 02974621; 02498613
Ramucirumab (VEGF)	Olaparib	I/II	Gastric	03008278
Bevacizumab (VEGF)	Niraparib	I/II	Ovarian	02354131
*PI3K/AKT/mTOR pathway*				
AZD5363 (PI3K)	Olaparib	I	Solid tumors	02338622
Everolimus (mTOR)	Niraparib	I	Breast, ovarian	03154281
*Other targeted therapies*				
Selumetinib	Olaparib	I	Solid tumors	03162627
AT13387 (Hsp90)	Olaparib	I	Ovarian and breast	02898207
Lapatanib (HER2)	Veliparib	I	Breast	02158507
*Hormonal therapy*				
Abiraterone	Olaparib	II	Prostate	01972217
Enzalutamide	Niraparib	I	Prostate	02500901
*Immunotherapy*				
*Anti-PD1*				
Nivolumab	Veliparib	I	Solid tumors, lymphoma	03061188
Pembrolizumab	Niraparib	I	Breast, ovarian	02657889
Nivolumab + platinum doublet	Veliparib	II	NSCLC	02944396
*Anti-PDL1*				
Durvalumab	Olaparib	II	Breast	03167619
Durvalumab + tremelimumab	Olaparib	I	Ovarian	02953457
Atezolizumab	Rucaparib	I	Gynaecological	03101280
Atezolizumab	Veliparib	II	Breast	02849496
